# Hahnemann’s Use of the Tincture

**Published:** 1888-10

**Authors:** Charles B. Gilbert

**Affiliations:** Washington, D. C.


### HAHNEMANN'S USE OF THE TINCTURE.

Editors Homœopathic Physician:—In the September number of the journal you have quoted a case treated by Hahnemann, and have there said: "A dose of this drug was given in a low potency, and the patient was well the next day." Hahnemann's prescription was exactly this: "As the woman was very robust, and as the forces of disease had affected her organism so painfully that she was not able to continue her work, and as, moreover, her vital powers were unimpaired, I gave her a full drop of the tincture of Bryonia * * ."

So it seems that he didn't give her a *potency* (so-called) at all, but the *crude drug.*

Let us have it exactly as it is.

Washington, D. C. Chas. B. Gilbert, M. D.

(Note.—Dr. Gilbert is entirely correct in saying Hahnemann gave the *tincture* of Bryonia in the case reported. In using the word *dilution* an unintentional mistake was made; there was no intention to mislead. But the whole truth as to Hahnemann's later views is that he regarded the use of the tincture as unwise and not to "be imitated." *Vide* foot-note, page 771, *Lesser Writings.*—E. J. L.)

